# Evaluation of antioxidant activity of *Malus domestica* fruit extract from Kashan area

**Published:** 2012

**Authors:** Sara Jelodarian, Abdolrasoul Haghir Ebrahimabadi, Ahmad Khalighi, Hossain Batooli

**Affiliations:** 1 *Faculty of Agriculture and Natural Sources, Science and Research Branch, Islamic Azad University, Tehran, I. R. Iran*; 2 *Essential Oils Research Institute, University of Kashan, Kashan, I. R. Iran*; 3 *Isfahan Research Center of Natural Resources, Kashan Station, Kashan, I. R. Iran*

**Keywords:** Apple, *Malus domestica*, Extract, Antioxidant activity, Total phenolic content

## Abstract

**Objective:** Antioxidants are considered as the main factors in the inhibition of unwanted oxidation reactions.

**Materials and Methods:** In this research the antioxidant potential of the fresh fruits of 4 cultivars (A to D) of *Malus domestica (M. domestica) *cultivated in the Kashan, Qamsar area was evaluated. The antioxidant activity of the samples were evaluated using two complementary antioxidant assays: 2, 2-diphenyl-1-picrylhydrazyl (DPPH) and *β*-carotene/linoleic acid tests and the results were compared with the synthetic standard antioxidant butylated hydroxytoluene (BHT).

**Results:** Total phenolic contents of the samples are also estimated by Folin-Ciocalteu's phenol test. In both DPPH *β*-carotene/linoleic acid tests in the concentration of 2 mg/ml, only samples from cultivar A showed moderate antioxidant activity with 63.92±0.42 and 6.02±0.03 inhibition percentages, respectively and other samples were weakly active.

**Conclusion:** The Folin-Ciocalteu's phenol test was also showed very little phenolic compounds for the fruits. In conclusion, weak antioxidant activity was estimated for the studied apple cultivars.

## Introduction

Free radicals are present in biological systems and may oxidize all the biological molecules present in our body, such as nucleic acids, proteins, lipids, initiating degenerative diseases (Cook et al., 1996 ▶; Harborne et al., 2000 ▶; Heim et al., 2002 ▶). Antioxidants are substances that neutralize free radicals and their negative effects. Antioxidants can inhibit or delay the oxidation of oxidizable substrates and this appears to be very important in the prevention of oxidative stress which is suggested as the leading cause of many oxidation related diseases (Bamoniri et al., 2010 ▶). 

Also antioxidants are substances that are able to prevent or retard the oxidation of lipids, proteins and DNA; and to protect the compounds or tissues from damage caused by oxygen or free radicals. Therefore, their health promoting effects reduce the risk of various diseases (Manach et al., 2004 ▶).Recently, antioxidant activity has been determined in many species of fruits, vegetables, herbs, cereals, sprouts and seeds (Kahkonen et al., 1999 ▶; Velioglu et al., 1998 ▶). Especial attention is paid to fruits, as rich sources of phenolic compounds (Kalt et al., 1999 ▶; Robards et al., 1999 ▶; Wang & Lin., 2000 ▶). Among others, the antioxidant properties of apple polyphenols have been extensively examined (Ju & Bramlage., 1999 ▶; Lu & Foo., 2000 ▶; Robards et al., 1999 ▶). Apples have the highest levels of antioxidant activity (Chinnici et al., 2004 ▶). Activity and concentration of antioxidants in fruit differ among cultivars, the part of the fruit, the growth stage and environmental conditions (Awad et al., 2001a ▶, b, c; Addie et al., 2001).

Apple fruit contain several health and sensory related constituents including dietary fiber, sugars, vitamins and phenolic compounds(Hagen et al., 2007 ▶).The antioxidant capacity of apple is mostly attributed to phenolic compounds such as ﬂavonoids and phenolic acids (Eberhardtet al., 2000 ▶; Lee et al., 2003 ▶).

*M. domestica* Borkh. is one of the most commonly consumed fruit worldwide (Shoji et al., 2004 ▶) and we collected samples named Hossain, Sayyed Babaeei, Shekareh and Golab, were randomly named as A, B, C and D, from Iran. 

These samples have been cultivated since most past times are medium in size with a circular shape. The yellow–pink skins are thin, rather wax-like, and the white ﬂeshes are soft, juicy, aromatic and sweet. Because of staying on the tree, the skin color of these 4 apple cultivars changes gradually and becomes red. Thus, the present research reports (1) the *in vitro* profile of the antioxidant activity of the fruit extracts using two complementary assays: DPPH radical and β-carotene linoleic acid tests; (2) the total phenolic content of the fruit extracts, expressed as gallic acid equivalents.

## Materials and Methods

**Fruit collection**

Fresh fruit samples from Hossain, Sayyed Babaeei, Shekareh and Golab apple cultivars were collected in the Kashan, Qamsar area in the June 2008 when the fruit had just been harvested.

**Extraction procedure**

Apples characterized by plant taxonomist, immediately transported to the laboratory, washed, dried, cut manually with a knife into small pieces, whole fruit except seeds extracts were obtained using a kitchen-type blender (Moulinex, France) and concentrated with a rotary evaporator.

**Solvents and chemicals**

2, 2-Diphenyl-1-picrylhydrazyl (DPPH) radical, β -carotene, linoleic acid, 2,6-di-tert-butyl-4-methylphenol (butylated hydroxytoluene, BHT) and gallic acid were procured from Sigma–Aldrich Chemie (Steinheim, Germany). Analytical grade methanol, ethanol, and dimethylsulfoxide (DMSO), HPLC grade chloroform, standard Folin–Ciocalteu’s phenol reagent, sodium carbonate, Tween 40, and all cultures media were obtained from Merck (Darmstadt, Germany). Ultra pure water was used for the experiment.

**Antioxidant activity DPPH radical scavenging**

The 2, 2-diphenyl-1-picrylhydrazyl (DPPH) radical assay usually involves hydrogen atom transfer reaction but, based on kinetic data, an electron transfer mechanism has also been suggested for this assay (; ). Radical scavenging activities of the plant essential oil and extract were determined using a published DPPH radical scavenging activity assay method (Sarker et al., 2006 ▶) with minor modiﬁcations.

Brieﬂy, stock solutions (10 mg/ml each) of the extracts and the synthetic standard antioxidant BHT were prepared in methanol. Dilutions are made to obtain concentrations ranging from 1 to 510^10 ^mg/ml. Diluted solutions (1 ml each) were mixed with 1 ml of a freshly prepared 80 µg/ml DPPH radical methanol solution and allowed to stand for 30 min in the dark at room temperature for any reaction to take place. Absorbance values of these solutions were recorded on an ultraviolet and visible (UV–Vis) spectrometer (Cintra 6, GBC, Dandenong, Australia) at 517 nm using a blank containing the same concentration of DPPH radicals. Inhibitions of DPPH radical in percent (I%) were calculated as follow (Gholivand et al., 2010 ▶): 

I% = [(A_blank_ - A_sample_)/A_blank_] × 100

In this research, dilution wasn’t performed due to low concentration of extracts and low inhibitory percentage. Where A_blank_ is the absorbance value of the control reaction (containing all reagents except the test compound) and A_sample _ is the absorbance values of the test compounds. The sample concentration providing 50% inhibition (half-maximal inhibitory concentration, IC_50_) was calculated by plotting inhibition percentages against concentrations of the sample. 

**β-Carotene/linoleic acid bleaching**

The β-carotene/linoleic acid test evaluates the inhibitory effect of a compound or a mixture on the oxidation of β-carotene in the presence of molecular oxygen (O_2_). Assay of the remained β-carotene gives an estimation of the antioxidant potential of the sample. The method described by Miraliakbari and Shahidi (), was used with slight modiﬁcations. A mixture of β-carotene and linoleic acid was prepared by adding together of 0.5 mg β-carotene in 1 ml chloroform (HPLC grade), 25 µl linoleic acid and 200 mg Tween 40. The chloroform was then completely evaporated under vacuum and 100 ml of oxygenated distilled water was subsequently added to the residue and mixed gently to form a clear yellowish emulsion. The essential oil, extract and BHT (positive control) were individually dissolved in methanol (2 g/l) and 350 µl volumes of each of them were added to 2.5 ml of the above emulsion in test tubes and mixed thoroughly. The test tubes were incubated in a water bath at 50 °C for 2 h together with a negative control (blank) contained the same volume of methanol instead of the extracts. The absorbance values were measured at 470 nm on an ultraviolet and visible (UV–Vis) spectrometer (Cintra 6, GBC, Dandenong, Australia). Antioxidant activities (inhibitions percentage, I%) of the samples were calculated using the following equation (Bamoniri et al., 2010 ▶):

I% = (A_-__β__carotene after 2-h assay_/A_initial __β__-carotene_) 100

Where A_-__β__carotene after 2-h assay_ is the absorbance values of β-carotene after 2 h assay remaining in the samples and A_initial __β__-carotene _is the absorbance value of β-carotene at the beginning of the experiments. All tests were carried out in triplicate and inhibition percentages were reported.

**Total phenolics**

Total phenolic constituents of extracts of 4 apple cultivars were determined by literature methods involving Folin–Ciocalteu’s phenol reagent and gallic acid standard (Slinkard & Singleton., 1977 ▶). A solution of the extract (0.1 ml) containing 1000 µg of the extract was pipetted into a 50 ml volumetric ﬂask, 46 ml distilled water and 1 ml Folin–Ciocalteu’s phenol reagent were added, and the ﬂask was thoroughly shaken. After 3 min, 3 ml of 2% Na_2_CO_3 _solution was added and the mixture was allowed to stand for 2 h with intermittent shaking. Absorbance values were measured at 760 nm. The same procedure was repeated for all the standard gallic acid solutions (0–1000 lg/0.1 ml) and a standard curve obtained with the following equation (Bamoniri et al., 2010 ▶):

Absorbance = 0.0012 × gallic acid (μg) + 0.0033

Total phenols of the extract, as gallic acid equivalent, was determined by using the absorbance value of the extract measured at 760 nm as input to the standard curve and the equation. Test was carried out in triplicate and gallic acid equivalent value was reported.

## Results

**DPPH**

DPPH radical scavenging activity potentials of fruit extract were evaluated for the assessment of their antioxidant capacities and compared with BHT (the standard commercial synthetic antioxidant). Among the above mentioned extracts, the best radical scavenging activity against DPPH was observed in cultivar A (63.92±0.42%) in the concentration of 2 mg/ml. The results obtained from 4 apple cultivars and BHT are presented in Table 1.

**Table 1:** DPPH radical scavenging activity (percentage ± SD) of 4 apple cultivars in the concentration of 2 mg/ml.

**Sample**	**Inhibition (%)**	
**A**	63.92 ± 0.42	
**B**	39.60 ± 0.75
**C**	19.99 ± 0.24	
**D**	43.16 ± 1.92	
**BHT** a	96.65 ± 0.15	

a In concentration of 0.5 mg/ml.

**β-Carotene/linoleic acid**

The potential of the plant to inhibit lipid peroxidation was evaluated using the β-carotene/linoleic acid bleaching test. In *β*-carotene/linoleic acid tests in the concentration of 2 mg/ml, only samples from cultivar A showed 6/015 ± 0/003 inhibition percentages. The results of 4 apple cultivars and standard (BHT) are presented in Table 2.

**Table 2:** Antioxidant activity of β-carotene/linoleic acid bleaching assay method (percentage ± SD) of 4 apple cultivars in the concentration of 2 mg/ml

**Sample**	**β-carotene bleaching (%)**
**A**	6.02** ± **0.03
**B**	4.24**± **0.56	
**C**	1.00** ± **0.05
**D**	3.16**± **0.08
**BHT**	96.40 **± **0.07

**Total phenolic constituents**

Total phenolic content of the plant extracts were determined using a colorimetric assay method based on Folin–Ciocalteu reagent reduction. 

The Folin-Ciocalteu's phenol test was also showed very little phenolic compounds for the fruits. The amounts of total phenols found in the fruit extracts are shown in Table 3.

**Table 3:** The contents of total phenol of 4 apple cultivars

**Sample**	**Total phenol contents (µg/mg)**
**A**	0 **±** 0
**B**	0 **±** 0
**C**	0 **±** 0
**D**	1.5 **±** 0.42

## Discussion

The measurement of the antioxidant capacity of food extracts and pure compounds is commonly performed using several methods. Each method relates to the generation or use of a different radical that is directly involved in the oxidative process, acting through a variety of mechanisms.Among the various assays, we selected the DPPH and β-Carotene/linoleic acid assays to determine the antioxidant activity of fruit extracts.

During DPPH radical test, the capacity of the samples to donate hydrogen atom and/or electron to this blue/purple stable radical and converting it to yellow diphenylpicrylhydrazine molecule was measured (Tepe et al., 2005 ▶). This reaction is used for measuring the ability of the extracts or pure molecules (such as BHT) to scavenge free radicals. Our results estimate a mild antioxidant potential for the cultivar A while other samples were weakly active.

Results of antioxidant test of 4 apple cultivars showed that none of 4 samples did not have high antioxidant properties at 2 mg/ml concentration but only cultivar A showed %64 inhibitory power.It is to be noted that the extracts were prepared with low concentration, therefore samples were not diluted.

These findings are in agreement with measured total phenolic contents of the samples (Drogoudi et al., 2008 ▶; Lata, 2007 ▶; D΄Abrosca et al., 2007 ▶; Tsao et al., 2005 ▶; Vieira et al., 2009 ▶). 

β-Carotene/linoleic acid test of 4 apple cultivars showed the same results as antioxidant test with the exception that cultivar A showed greater inhibitory power (%6) compared to DPPH procedure.

This finding is in contradiction with findings of Garcia et al., 2009; Lata et al., 2009; Lee et al., 2003; Bandoniene and Murkovic., 2002; Kondo et al., 2002; which might be due to different cultivars they have selected under different climatic conditions.

The basic structure of the phenols and other structural factors play a fundamental role in the mechanism by which these compounds are able to scavenge free radicals (Sadeghipour et al., 2005 ▶). As underlined also by others (Lata et al., 2009 ▶; Lata, 2008 ▶), it is difficult to compare the content of apple phenolic among different studies, as many variations can be principally caused by different growth period, geographic location, storage type, genetic diversity and many other factors.

The results, expressed as gallic acid equivalents, were 0 µg/mg and 1.5±0.6 for the extracts of apples, respectively.

These values are comparable to the values reported in the literature for other apple cultivars, such as Golden Delicious, Stark Delicious, Mora, Nesta, Panaia-red and Ruggine (Iacopini et al., 2009 ▶). Phenolic compounds normally play main role in the antioxidant activity of the plant extracts, thus, low DPPH antioxidant activity of our samples may be related to their negligible total phenolic compounds contents. Folin–Ciocalteu test showed that there is low percentage of phenolic compounds in all samples which is in accordance with antioxidant tests.

Overall conclusions was that all samples did not show high antioxidant power and only cultivar A showed higher antioxidant power, which might be due to the presence of phenolic compounds.

## Conclusions

Natural products, especially those produced by edible and medicinal plant species, are currently under special interest as food additive due to their safety, usefulness and accessibility. In this study, we have focused on antioxidant activity and total phenolic compounds of apples. Our results conclude that the phenolic content, the radical-scavenging and antioxidant properties of old local apple varieties demonstrate that these new cultivars could be a good source of phytochemicals, bioactive compounds with important protective properties. These local apple cultivars could be also considered as an important source of genes for apple breeding program and for the production of value added apple cultivar. Thus, further studies on local and ancient varieties for determining of their biological potentials have are highly recommended.
